# The influence of gastric atrophy on *Helicobacter pylori* antibiotics resistance in therapy-naïve patients

**DOI:** 10.3389/fmicb.2022.938676

**Published:** 2022-09-23

**Authors:** Elisabetta Goni, Ina Tammer, Kerstin Schütte, Cosima Thon, Dörthe Jechorek, Ujjwal Mukund Mahajan, Riccardo Vasapolli, Lukas Macke, Benedikt Aulinger, Michael Selgrad, Alexander Link, Peter Malfertheiner, Christian Schulz

**Affiliations:** ^1^Department of Medicine II, University Hospital, LMU Munich, Munich, Germany; ^2^Otto-von-Guericke University Hospital, Institute of Medical Microbiology, Magdeburg, Germany; ^3^Department of Internal Medicine and Gastroenterology, Niels-Stensen-Kliniken, Marienhospital, Osnabrück, Germany; ^4^Department of Gastroenterology, Hepatology, and Endocrinology, Hannover Medical School, Hannover, Niedersachsen, Germany; ^5^Department of Gastroenterology, Hepatology and Infectious Diseases, Otto-von-Guericke University Hospital, Magdeburg, Germany; ^6^Department of Pathology, Otto-von-Guericke-University Magdeburg, Magdeburg, Germany; ^7^Deutsches Zentrum für Infektionsforschung (DZIF), Partner Site Munich, München, Germany; ^8^Department of Internal Medicine, Klinikum Fuerstenfeldbrueck, Fuerstenfeldbrueck, Germany; ^9^Department of Internal Medicine I, University Hospital of Regensburg, Regensburg, Germany

**Keywords:** antibiotic resistance rate, antibiotic susceptibility testing, antibiotic stewardship, updated treatment strategies, *Helicobacter pylori* infection, chronic atrophic gastritis, intestinal metaplasia

## Abstract

**Background:**

Antibiotic susceptibility of *Helicobacter pylori* to antibiotics may vary among different niches of the stomach. The progression of chronic *H. pylori* gastritis to atrophy changes intragastric physiology that may influence selection of resistant strains.

**Aim:**

To study the antibiotic resistance of *H. pylori* taking the severity of atrophic gastritis in antrum and corpus into account.

**Methods:**

*Helicobacter pylori*-positive patients (*n* = 110, *m* = 32, mean age 52.6 ± 13.9 years) without prior *H. pylori* eradication undergoing upper gastrointestinal (GI) endoscopy for dyspeptic symptoms were included in a prospective study. Patients were stratified into three groups depending on the grade of atrophy: no atrophy (OLGA Stage 0), mild atrophy (OLGA Stage I–II) and moderate/severe atrophy (OLGA Stage III–IV). Two biopsies each from the antrum and the corpus and one from the angulus were taken and assessed according to the updated Sydney system. *H. pylori* strains were isolated from antrum and corpus biopsies and tested for antibiotic susceptibility (AST) for amoxicillin, clarithromycin, metronidazole, levofloxacin, tetracycline, and rifampicin by the agar dilution methods. A Chi-square test of independence with a 95% confidence interval was used to detect differences in the proportion of patients with susceptible and resistant *H. pylori* strains.

**Results:**

Among 110 patients, primary clarithromycin resistance (R) was 30.0%, both in the antrum and corpus; metronidazole resistance accounted for 36.4 and 34.5% in the antrum and corpus; and levofloxacin was 19.1 and 22.7% in the antrum and corpus, respectively. Resistance rates to amoxicillin, tetracycline, and rifampicin were below 5%. Dual antibiotic resistance rate was 21.8%, and triple resistance rate was 9.1%. There was a significant difference in the resistance rate distribution in antrum (*p* < 0.0001) and corpus (*p* < 0.0001). With increasing severity of atrophy according to OLGA stages, there was a significant increase in clarithromycin-R and metronidazole-R.

**Conclusion:**

In treatment-naïve patients, antibiotic resistance and heteroresistance were related to the severity of atrophy. The high clarithromycin resistance in atrophic gastritis suggests that *H. pylori* antibiotic susceptibility testing should always be performed in this condition before selecting the eradication regimen.

## Introduction

*Helicobacter pylori* treatment regimens require the combination of an acid suppressant with two and up to three antibiotics (Sugano et al., 2015; Malfertheiner et al., 2017).

Resistance (R) to commonly used antibiotics (e.g., clarithromycin, metronidazole, and levofloxacin) has dramatically increased and is the main cause of treatment failure in *H. pylori* eradication. The overuse of these antibiotics for other indications (Chey et al., 2017; Fischbach et al., 2017; Malfertheiner et al., 2017) is the most likely explanation for the increasing antibiotic resistance (Austin et al., 1999; Canton and Morosini, 2011; Tacconelli et al., 2018). According to the World Health Organization (WHO), clarithromycin-resistant *H. pylori* is among bacteria with high priority for developing new antibiotics (Tacconelli et al., 2018). Important measures are undertaken by national and international networks for shriveling local, regional and national resistance development (European Study Group on Antibiotic Susceptibility of Helicobacter pylori, 1992; Glupczynski et al., 2001; Megraud et al., 2013; Wuppenhorst et al., 2014). Antibiotic stewardship (ABS) is advocated to better handle the emerging resistance by adopting therapies based on antibiotic susceptibility testing (AST).

In current practice, *H. pylori* eradication regimens in naïve patients are mostly prescribed empirically. A minority of patients undergoes AST before receiving first-line eradication therapy. In a recent European multicentric surveillance study (Hp-EuReg) only 11.1% of 21,533 underwent AST before first-line eradication therapy (Nyssen et al., 2021).

An issue to consider when using a single biopsy for AST is the possibility of synchronous presence of resistant and susceptible strains in the same individual (interniche heteroresistance), a phenomenon reported in 10–20% of cases. Gastric luminal and mucosal factors related to the degree of inflammation, grade of atrophy and gastric pH might interfere with bacterial metabolism and change drug pharmacokinetics. *H. pylori* gastritis is the prototypic environmental, not self-limiting gastritis. *H. pylori* infection leads to a cytotoxic damage of the resident glandular population (i.e., oxyntic glands in the corpus/fundus; mucosecreting glands in the antral mucosa) and it can progress to a loss of native glandular units (atrophy)” (Rugge et al., 2019). These changes in the gastric histology pattern and, consequently, in the gastric physiology (i.e., acid secretion, achlorhydria) influence the bioavailability of antibiotics used in eradication regimens (Mégraud, 2004; Suerbaum and Josenhans, 2007; Wueppenhorst et al., 2009; Mascellino et al., 2017; Ailloud et al., 2019; Kocsmár et al., 2021). Variations in gastric pH can alter the solubility or chemical stability of molecules such as beta-lactams, macrolides, and some azoles; consequently, the bioavailability of these drugs can be reduced if the gastric pH is raised, e.g., by PPI therapy or loss of acid-producing cells. Antibiotics such as tetracycline or fluoroquinolones have reduced bioavailability due to chelation from bi- and tri-valent cations (Kramer et al., 1978; Rugge et al., 2007; Mazzei, 2011). Regarding clarithromycin, it has very poor solubility at neutral intestinal pH, but much better solubility under acidic conditions due to amine protonation. The improved solubility in an acidic environment is based on the poor chemical stability of clarithromycin that is quite labile toward acid-catalyzed degradation (Capelle et al., 2010; Pereira et al., 2013).

The aims of our study are (a) to assess the primary antibiotic resistances in the different stages of severity of chronic atrophic gastritis, and (b) to analyze the relative prevalence of primary resistance in distinct anatomical sites of the stomach. The analysis included AST to amoxicillin, clarithromycin, metronidazole, levofloxacin, tetracycline and rifampicin.

## Materials and methods

### Ethical statement

All investigations were performed at the Department of Gastroenterology, Hepatology, and Infectious Diseases at Otto-von-Guericke University Magdeburg (Germany) from 2011 to 2015. The study was approved by the local Ethics Committee (IRB number 80/11, Otto-von-Guericke University Magdeburg). The study protocol was conducted according to the Declaration of Helsinki and Good Clinical Practice. All study participants provided written informed consent. This work was supported in part by a grant from the BMBF (BMBF-0315905D) within the ERA-Net PathoGenoMics project. Evaluation of data was performed in cooperation with the Medical Department 2 of the Ludwig Maximillian University Munich, Germany.

### Study design

One hundred ten *H. pylori* infected therapy-naïve patients requiring upper gastrointestinal (GI) endoscopy for dyspeptic symptoms were enrolled prospectively from 2011 to 2015. The following exclusion criteria were applied: previous *H. pylori* eradication therapies, operated stomach, upper abdomen irradiation, immunosuppressive therapy, oral anticoagulation, and any antibiotic therapy within the last 2 weeks before entering the study. Ongoing or prior proton pump inhibitor (PPI) therapy was not an exclusion criterion as many patients were already treated with PPI before being appointed for upper endoscopy.

### Methods

Standard video gastroscopes (GIF Q145, GIF 160, and GIF Q180 HD; Olympus Medical, Hamburg, Germany) and standard oval fenestrated cup forceps with a needle (Olympus SwingJaw 2.8 mm FB-240 K_A, Olympus Medical, Hamburg, Germany) were used.

Two biopsies each from the antrum and the corpus and one from the angulus were obtained for the updated Sydney system (lesser and larger curvature of the antrum at 3 cm distance from the pylorus, larger and lesser curvature of the middle corpus). These were immediately fixed in buffered formalin for histopathological assessment. According to OLGA, the degree of atrophic gastritis was staged (Cederbrant et al., 1993; Megraud and Lehours, 2007). One biopsy from the antrum and one biopsy from the corpus were taken for the *H. pylori* culture and stored immediately in Portagerm pylori^®^ tubes (bioMerieux, France). According to the updated Sydney system, a histopathological assessment of the gastric mucosa was performed. Sections were stained with hematoxylin and eosin, PAS staining technique and modified Giemsa to diagnose *H. pylori*.

The culture was performed on a Columbia-agar-based medium that contained 10 vol% washed human erythrocytes and 10 vol% heat-inactivated horse serum (purchased from the NRZ Nationale Referenzzentrum Helicobacter Freiburg, Germany) without and with an antibiotic supplement (vancomycin 10 mg/ml, nystatin 1 mg/ml, and trimethoprim 5 mg/ml) for suppressing the overgrowth of the oral flora. Incubation of the plates was performed under microaerophilic conditions at 37°C with CampyGen^™^ gasbags (Oxoid, Germany), and examination was done every 2–3 days for up to 10 days. Identification of *H. pylori* was performed by typical morphology on Gram stain and positive urease, oxidase and catalase tests (Glupczynski et al., 1991).

Susceptibility testing to amoxicillin, metronidazole, clarithromycin, tetracycline, levofloxacin and rifampicin was performed with the ETEST method (bioMerieux, France) on Iso-Sensitest agar with 10 vol% defibrinated horse blood (Oxoid, Germany; Selgrad et al., 2014; Bluemel et al., 2020). The ETEST can detect antibiotic-resistant sub-populations. In this study, agar plates were treated with suspensions of *H. pylori* after adjustment to turbidity approximately equal to that of a McFarland standard No. 3. The antibiotics’ minimum inhibitory concentrations (MICs) were determined after 3 days of incubation or until the inhibition zone became visible.

The EUCAST criteria were applied for all antibiotic substances tested within this study (European Committee on Antimicrobial Susceptibility Testing). Breakpoint tables for interpretation of MICs and zone diameters for *H. pylori* Version 2.0, 2012^1^ were used. A resistant isolate was defined if the MIC was above the following breakpoints (R): amoxicillin 0.125 mg/l, tetracycline and levofloxacin > 1 mg/l, clarithromycin > 0.5 mg/l, rifampicin > 1 mg/l, and metronidazole > 8 mg/l (EUCAST, 2018).

### Statistical analysis

The Chi-square test of independence with a 95% confidence interval was used to detect differences in the proportion of patients with susceptible and resistant *H. pylori* strains. *p*-values were considered significant if *p* < 0.05. Subsequently, Cramér’s *V* was estimated for the effect size for the Chi-square test of independence. Cramér’s *V* determines the degree of associations between two categories. All analyses were performed in R (version R-4.0.4)^2^ and R-studio (version 1.3.9.59; R-studio, Boston, MA, United States).

## Results

One hundred ten *H. pylori*-infected patients were included in the analysis (*n* = 110, *m* = 32, mean age 52.6 ± 13.9 years). None of the patients included in the study had been previously treated for *H. pylori* infection.

Overall, 74 patients (*n* = 74, 68.5%) had one or more resistances in the antrum or the corpus; a dual resistance in both antrum and corpus was observed in 64 patients (58%). The single, double, triple and quadruple resistance rates in antrum and in corpus according to OLGA staging are summarized in Table 1. In the antrum, we found resistance to amoxicillin only in one patient (0.9%), to clarithromycin in 33 patients (30.0%), to metronidazole in 40 patients (36.4%), to levofloxacin in 21 patients (19.1%) and to rifampicin in three patients (2.7%). In one patient (0.9%), we observed resistance to tetracycline in the antrum. A similar distribution in the antibiotic resistance was shown in the corpus: resistance to amoxicillin was present only in one patient (0.9%), to clarithromycin in 33 patients (30.0%), to metronidazole in 38 patients (34.5%), to levofloxacin in 25 patients (22.7%) and to rifampicin in four patients (3.6%). One patient had resistance against tetracycline in the corpus (0.9%). In both the antrum and/or corpus, a dual antibiotic resistance was detected in 24 patients (21.8%) and a triple resistance in ten patients (9.1%). Considering the correlation among resistance, age and sex, we found a statistically significant correlation between antibiotic resistance and age of the patients. Resistances were not influenced by sex of patients.

**Table 1:** Single, double, triple and quadruple resistance in antrum and corpus, according to the severity of atrophy.

OLGA Stage	ANTRUM			
	Single resistance	Double resistance	Triple resistance	Quadruple resistance
No atrophy (OLGA Stage 0) *n* = 46	12 (26.1%)	13 (28.3%)	2 (4.3%)	0
Mild Atrophy (OLGA Stage I–II) *n* = 40	4 (10.0%)	8 (20.0%)	1 (2.5%)	1 (2.5%)
Moderate/severe atrophic gastritis (OLGA Stage III–IV) *n* = 24	7 (29.2%)	3 (12.5%)	2 (8.3%)	0
CORPUS				
No atrophy (OLGA Stage 0) *n* = 46	11 (24.0%)	11 (24.0%)	2 (4.3%)	0
Mild Atrophy (OLGA Stage I–II) *n* = 40	13 (32.5%)	8 (20.0%)	4 (10.0%)	1 (2.5%)
Moderate/severe atrophic gastritis (OLGA Stage III–IV) *n* = 24	8 (33.3%)	4 (16.7%)	3 (12.5%)	0

According to the OLGA Staging System, patients were stratified into three groups: no atrophy (OLGA 0, *n* = 46, Group 1), mild atrophic gastritis (OLGA Stage I–II, *n* = 40, Group 2), and moderate/severe atrophic gastritis (OLGA Stage III–IV, *n* = 24, Group 3). The study population characteristics are summarized in Table 2.

**Table 2:** Study population characteristics (*n* = 110).

*Helicobacter pylori* therapy naïve patients (*n* = 110) Overall resistance rate *n* = 74/110 (67.3%)	
No atrophy (OLGA Stage 0) *n* = 46	
Sex	Males *n* = 14
Age (y.o ± SD)	47.7 ± 11.8
Resistance rate (overall)	30 (65.2%)
Mild atrophy (OLGA Stage I–II) *n* = 40	
Sex	Males *n* = 9
Age (y.o ± SD)	48.2 ± 13.8 years
Resistance rate (overall)	28 (70.0%)
Moderate/severe atrophic gastritis (OLGA Stage III–IV) *n* = 24	
Sex	Males *n* = 9
Age (y.o ± SD)	54.9 ± 14.0 years
Resistance rate (overall)	16 (66.7%)

y.o: years old; SD: standard deviation.

*H. pylori* was detected in the antrum of 96.2% of patients, in the corpus of 92.5% of patients, and both in antrum and corpus in 88.7% of the patients.

Among patients included in Group 1 (no atrophy OLGA 0, *n* = 46, 14 males, mean age 47.7 ± 11.8 years), 30 patients had at least one antibiotic resistance (overall resistance rate 65.2%) and 26 patients (86.7%) were observed having a dual resistance both in the antrum and in the corpus. Resistance against clarithromycin was found in the antrum of 16 patients (34.8%) and the corpus of 15 patients (32.6%). Fourteen patients showed a dual resistance for clarithromycin in both antrum and corpus. Resistance against metronidazole was demonstrated in 21 patients in the antrum (45.6%) and 20 patients in the corpus (43.5%). Resistance against levofloxacin was found in eight patients (17.4%) in the antrum, nine patients in the corpus (19.6%) and seven patients in both antrum and corpus. Resistance against rifampicin was observed in one patient in both antrum and corpus (2.2%). Resistance against amoxicillin and tetracycline was not detected in the antrum and the corpus.

The single, double, triple and quadruple resistance rates in Group 1 in antrum and in corpus are summarized in Table 1.

Forty patients were included in Group 2 (mild atrophic gastritis OLGA I-II, n = 40, 9 males, mean age 48.2 ± 13.8 years). Of those, 28 patients had at least one antibiotic resistance (overall resistance rate 70%) and 19 patients (67.8%) were observed a dual resistance both in the antrum and in the corpus. Resistance against clarithromycin was found in the antrum of ten patients (25.0%) and the corpus of 12 patients (30.0%). Nine patients presented with a dual resistance for clarithromycin both in the antrum and corpus. Resistance against metronidazole was demonstrated in 14 patients in the antrum (35.0%) and 17 patients in the corpus (42.5%). Resistance against levofloxacin was found in eight patients (20.0%) in the antrum, in 11 patients (27.5%) in the corpus and in six patients (15.0%) in both antrum and corpus. Resistance against rifampicin was observed in two patients in the antrum (5.0%) and three in the corpus (7.5%). No resistance against amoxicillin and tetracycline was detected both in the antrum and in the corpus in Group 2. The single, double, triple and quadruple resistance rates in Group 2 in antrum and in corpus are reported in Table 1.

Twenty-four patients were diagnosed with moderate-to-severe atrophic gastritis (OLGA Stage III–IV, *n* = 24, 9 males, mean age 54.9 ± 13 years). *H. pylori* was detected in 96.2% of patients in the antrum, 92.5% in the corpus and 88.7% in both antrum and corpus. Sixteen patients had at least one antibiotic resistance (overall resistance rate 66.7%) and ten patients (62.5%) were observed with a dual resistance both in the antrum and in the corpus.

Resistance against clarithromycin was found in seven patients in the antrum (36.8%), six patients in the corpus (28.6%), and a dual resistance was present in the antrum and the corpus of five patients. Resistance against metronidazole was demonstrated in five patients in the antrum (26.3%), 11 patients in the corpus (52.4%), and five patients (20.8%) in both antrum and corpus. Resistance against levofloxacin was found only as dual resistance—in both antrum and corpus—in five patients (26.3%). One patient had resistance against amoxicillin (5.3%) and one against tetracycline (5.3%). Resistance against rifampicin was not detected in the antrum or the corpus. The single, double, triple and quadruple resistance rates in Group 3 in antrum and in corpus according to OLGA staging are summarized in Table 1.

**Figure 1:**
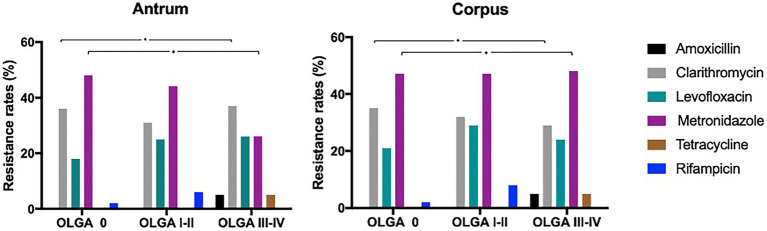
Distribution of resistance rate to amoxicillin, clarithromycin, metronidazole, levofloxacin, tetracycline, and rifampicin according to the different grade of atrophic gastritis (OLGA Stage 0, OLGA I–II, OLGA Stage III–IV) in the antrum and corpus. OLGA: Operative Link for Gastritis Assessment. The Chi-square test of independence with a 95% confidence interval was used to detect differences in the proportion of patients with susceptible and resistant *H. pylori* strains. ^*^Clarithromycin (Antrum: *X*^2^ Pearson (4) = 1.82, *p* = 0.03, V Cramer = 0.00, Cl 95% 0.00–1.00; corpus: *X*^2^ Pearson (4) = 3.48, *p* = 0.01, V Cramer = 0.00, Cl 95% 0.00–1.00) ^*^Metronidazole (Antrum: *X*^2^ Pearson (2) = 4.95, *p* = 0.0098, V Cramer = 0.18, Cl 95% 0.00–1.00; Corpus: *X*^2^ Pearson (2) = 0.62, *p* = 0.02, V Cramer = 0.00, Cl 95% 0.00–1.00).

The overall antibiotic resistance rate among the three groups (OLGA Stage 0 versus OLGA Stage I–II versus OLGA Stage III–IV) is summarized in Figure 1.

Overall, there was a statistically significant difference in the resistance rate distribution to amoxicillin, clarithromycin, metronidazole, levofloxacin, tetracycline, and rifampicin in antrum (*X*^2^ Pearson (10) = 112.89, *p* = 1.39*e*–19, V Cramer = 0.30, Cl 95% 0.23–1.00; Figure 1) and in corpus (*X*^2^ Pearson (10) = 131.99, *p* = 1.83*e*–23, V Cramer = 0.32, Cl 95% 0.25, 1.00; Figure 1).

When comparing OLGA Stage 0 versus OLGA Stage I–II versus OLGA Stage III–IV, our data showed a statistically significant difference in the resistance to clarithromycin (*X*^2^ Pearson (4) = 1.82, *p* = 0.03, V Cramer = 0.00, Cl 95% 0.00–1.00), to metronidazole (*X*^2^ Pearson (2) = 4.95, *p* = 0.0098, V Cramer = 0.18, Cl 95% 0.00–1.00) in antrum, as well as to clarithromycin (*X*^2^ Pearson (4) = 3.48, *p* = 0.01, V Cramer = 0.00, Cl 95% 0.00–1.00) and to metronidazole (*X*^2^ Pearson (2) = 0.62, *p* = 0.02, V Cramer = 0.00, Cl 95% 0.00–1.00) in corpus (Figure 1).

## Discussion

In this study the prevalence of *H. pylori* resistance to clarithromycin and metronidazole increases in parallel to the progression in severity of atrophic gastritis. This is not the case for levofloxacin across all stages of atrophic gastritis. Amoxicillin, tetracycline, and rifampicin resistance were very low, therefore without clinical significance.

The main point of our study is the analysis of susceptibility to key antibiotics used in eradication regimens at different stages of chronic gastritis along with the OLGA staging system. In this therapy-naïve cohort, there is a significant difference in the resistance rate distribution to clarithromycin, metronidazole and levofloxacin, in antrum and corpus, along the cascade of atrophic gastritis.

According to the grade of gastric atrophy, our results showed a significant increase in the prevalence of resistance as well as heteroresistance in general.

When comparing OLGA Stage 0 versus OLGA Stage I–II versus OLGA Stage III–IV, the clarithromycin and metronidazole resistance rates were often different between the antrum and corpus. This was less frequent the case with respect to levofloxacin (18.6–23.8% of resistance). However, in our study, an increased overall resistance rate to levofloxacin compared to earlier studies was observed (Glupczynski et al., 2001; Mégraud, 2004; Megraud et al., 2013; Kocsmár et al., 2021). The same trend was shown to a lesser degree for rifampicin as a reserve antibiotic based on antibiotic susceptibility testing (AST) results. The variations in gastric pH and consequently in the antibiotics bioavailability could explain the statistical difference in the antibiotic resistance rate along the cascade of atrophic gastritis (Kramer et al., 1978; Rugge et al., 2007; Capelle et al., 2010; Mazzei, 2011; Pereira et al., 2013). Furthermore, atrophic gastritis is the long-term expression of *H. pylori* infection and this time-phenotype correlation very likely results in higher exposure to antimicrobial therapies. These factors may all play a crucial role and interact in *H. pylori* resistance development that can occur during infection, resulting in spontaneous mutations (Correa et al., 1975; McGowan Jr., 1983; Harbarth et al., 2001; Albrich et al., 2004; Committee Opinion No. 465, 2010; Ledger and Blaser, 2013; Moss, 2017). According to the current guidelines, due to the increasing number of metronidazole-resistant *H. pylori* strains, metronidazole in first-line treatment is only selectively recommended in Europe (Malfertheiner et al., 2017). Regarding clarithromycin resistance, current guidelines recommend the exclusion of clarithromycin resistance before use or empiric medication depending on local resistance rates (Malfertheiner et al., 2017). However, standardized diagnostic and therapeutic algorithms based on AST in managing *H. pylori* infection could improve the eradication rates and minimize the development of antibiotic resistance worldwide. The WHO recognized the challenge of growing antibiotic resistance rates of *H. pylori* and added this bacterium to 12 pathogens for which new antibiotics are urgently needed (Tacconelli et al., 2018). In several studies, susceptibility-guided therapies showed improved results compared to empirical antibiotic regimes (Wenzhen et al., 2010; Lopez-Gongora et al., 2015; Malfertheiner et al., 2017).

Regarding heteroresistances, in our cohort, patients with mild gastritis (OLGA I–II) had a heteroresistance rate of 45.5%, compared to 81.2% for patients with advanced gastritis (OLGA III–IV). To our knowledge, this is the first study correlating the *H. pylori* antibiotic resistance rate in therapy-naïve patients with the severity of atrophic gastritis; for this reason, limited data on this topic in literature are available. The biological plausibility of this heterogeneous distribution of antibiotic resistance according to the severity of the atrophy remains unclear, but possible explanations are a higher selection pressure on *H. pylori* in severely damaged mucosa and changes in gastric acidity (Kramer et al., 1978; Rugge et al., 2007; Capelle et al., 2010; Mazzei, 2011; Pereira et al., 2013). Furthermore, in our study, more than 60% of antrum samples and more than 70% of corpus samples demonstrated at least one resistance and thus justified the recommendation of early resistance testing, preferably during the first invasive procedure used in the diagnostic workup of patients. Concerning the significant increase and the development of gastric preneoplastic lesions, the subgroup of patients suffering from atrophic gastritis should be highlighted. Several studies demonstrated different antibiotic susceptibilities between antrum and corpus in eradication-naïve and eradication-failed subjects. Various methods are established for *in vitro* susceptibility testing. PCR-based testing opens the AST for additional materials, such as feces and embedded biopsies. This evidence of interniche heteroresistance of *H. pylori* represents a relevant problem in clinical practice and the concomitant presence of *H. pylori* strains with different resistance spectrums in the same patient is likely to cause treatment failure and increase resistant strains’ selection; consequently our findings contribute to the ongoing debate regarding the timepoint of AST.

Our study has some limitations. Like most studies in this field, our work was based on the patient history of previous antibiotic exposure, which is not always reliable. The enrollment of subjects in only one tertiary referral center does not allow for the interpretation of quantitative resistance rates, but the relationship between the stratified groups following histopathological results is useful (Kim et al., 2003; Mégraud, 2004; Rimbara et al., 2005; Ayala et al., 2011).

In conclusion, our results report the different distributions of resistance prevalence according to the grade of gastric atrophy. Our findings underline the importance of early AST in the therapeutic algorithm of *H. pylori* infection, especially in patients with moderate/severe atrophy.

## Data availability statement

The raw data supporting the conclusions of this article will be made available by the authors, without undue reservation.

## Ethics statement

The studies involving human participants were reviewed and approved by Otto-von-Guericke University Magdeburg. IRB number 80/11. The patients/participants provided their written informed consent to participate in this study.

## Author contributions

CS, EG, KS, and PM: conceptualization and writing—original draft preparation. CS, IT, and DJ: methodology. UM: formal analysis. BA and LM assisted with data analysis. EG, CS, AL, MS, and KS: investigation. EG, RV, KS, AL, and MS: patients’ enrollment. CT and AL: biobanking. EG, CS, and KS: data curation. PM and CS: supervision. CS: project administration. PM, KS, and CS: funding acquisition. Each author has approved the submitted version and agrees to be personally accountable for the author’s own contributions and for ensuring that questions related to the accuracy or integrity of any part of the work, even ones in which the author was not personally involved, are appropriately investigated, resolved, and documented in the literature. Each author contributed to the conception, design of the work, acquisition, and analysis and interpretation of data. All authors contributed to the article and approved the submitted version.

## Conflict of interest

The authors declare that the research was conducted in the absence of any commercial or financial relationships that could be construed as a potential conflict of interest.

## Publisher’s note

All claims expressed in this article are solely those of the authors and do not necessarily represent those of their affiliated organizations, or those of the publisher, the editors and the reviewers. Any product that may be evaluated in this article, or claim that may be made by its manufacturer, is not guaranteed or endorsed by the publisher.

## Footnotes

^1^ http://www.eucast.org

^2^ https://www.r-project.org/
